# Supplementary figures and images for: Systemic translocation of Staphylococcus aureus promotes autoimmunity: implications in autoantibody-mediated poor immune reconstitution from antiretroviral therapy in HIV
Source: J Virol. 2026 Apr 3;100(5):e01965-25. doi: 10.1128/jvi.01965-25 (PMC13185582; doi:10.1128/jvi.01965-25)

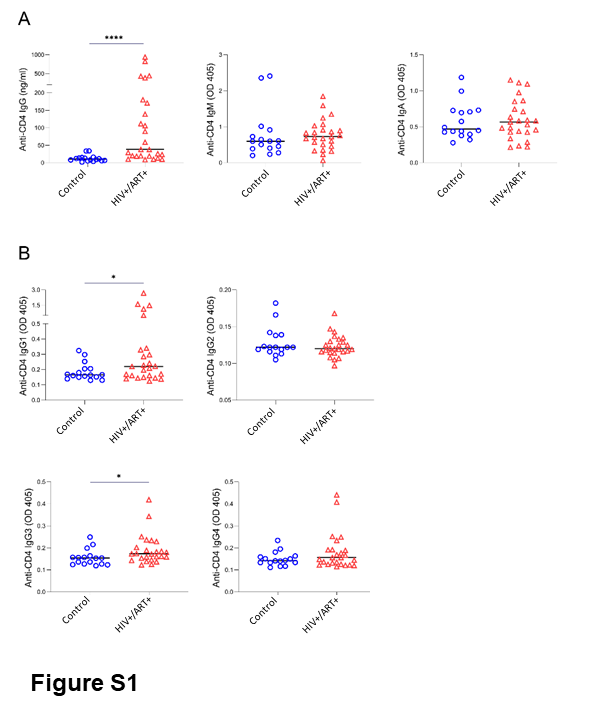

Supplement: Figure S1 — Elevated plasma anti-CD4 IgG1 and IgG3 subclasses in PWH on ART. [file jvi.01965-25-s0002.tif]

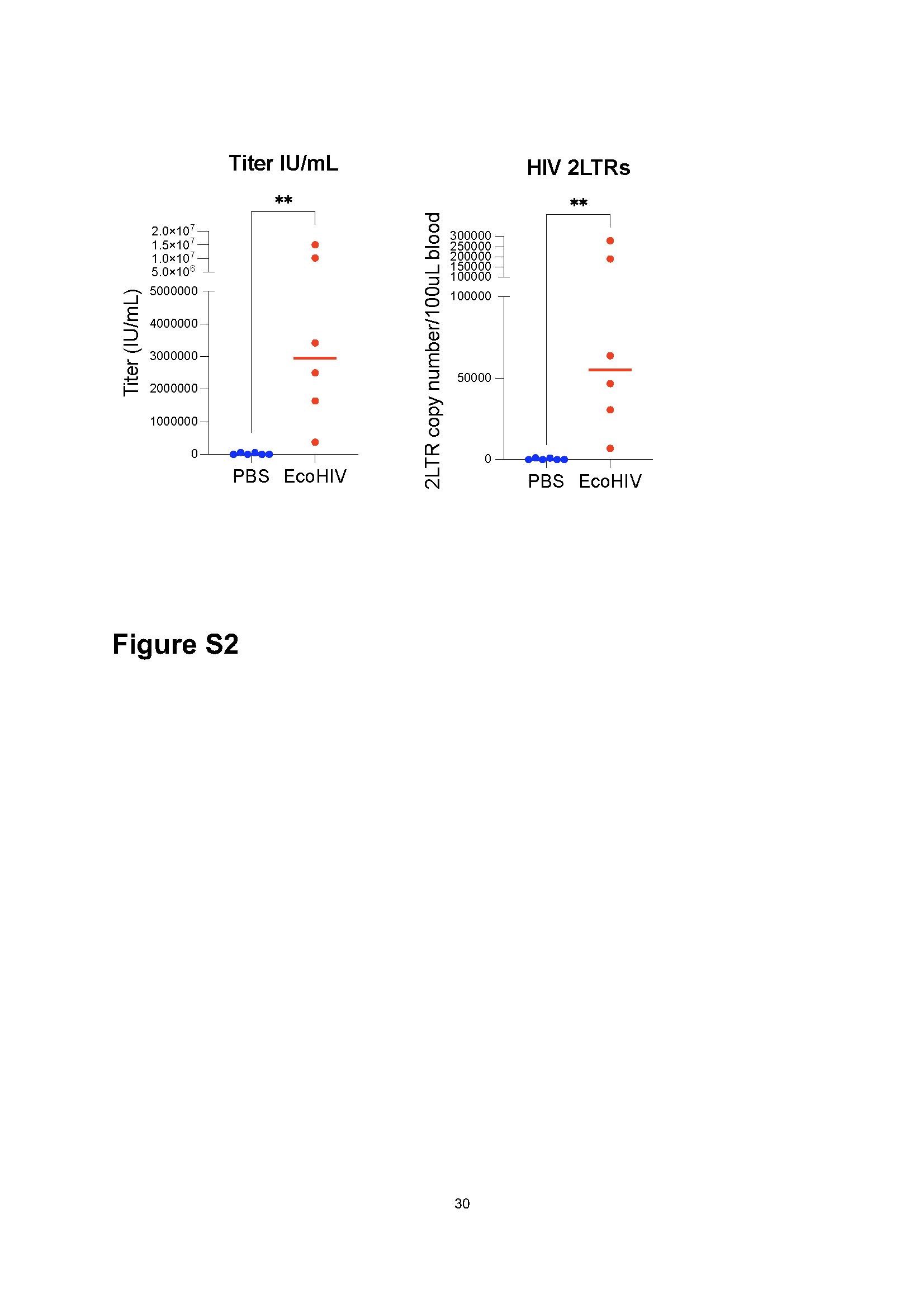

Supplement: Figure S2 — Confirmation of established EcoHIV infection in mice. [file jvi.01965-25-s0003.tiff]

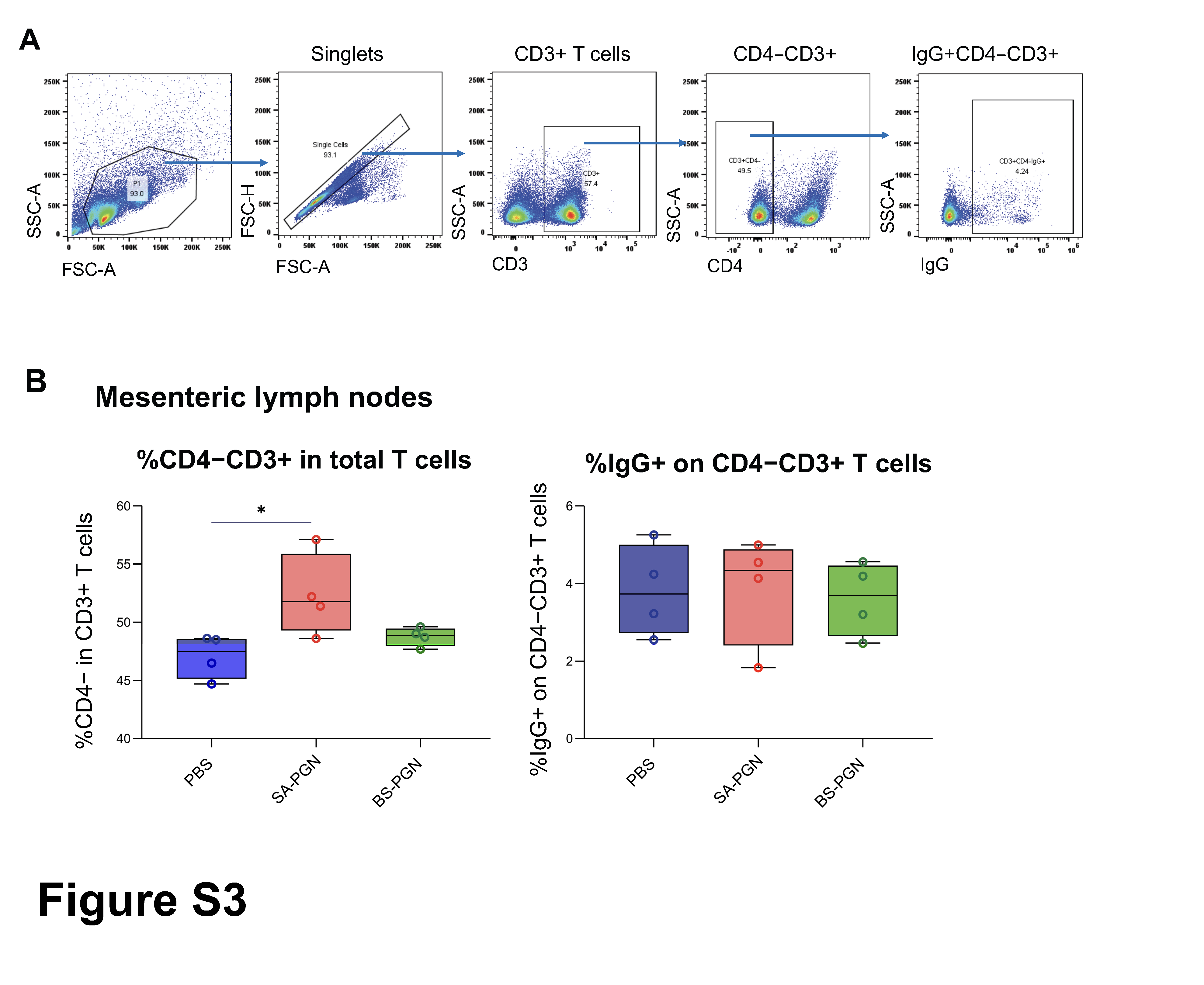

Supplement: Figure S3 — S. aureus PGN does not affect gut CD4−CD3+ T cells in vivo. [file jvi.01965-25-s0004.tiff]

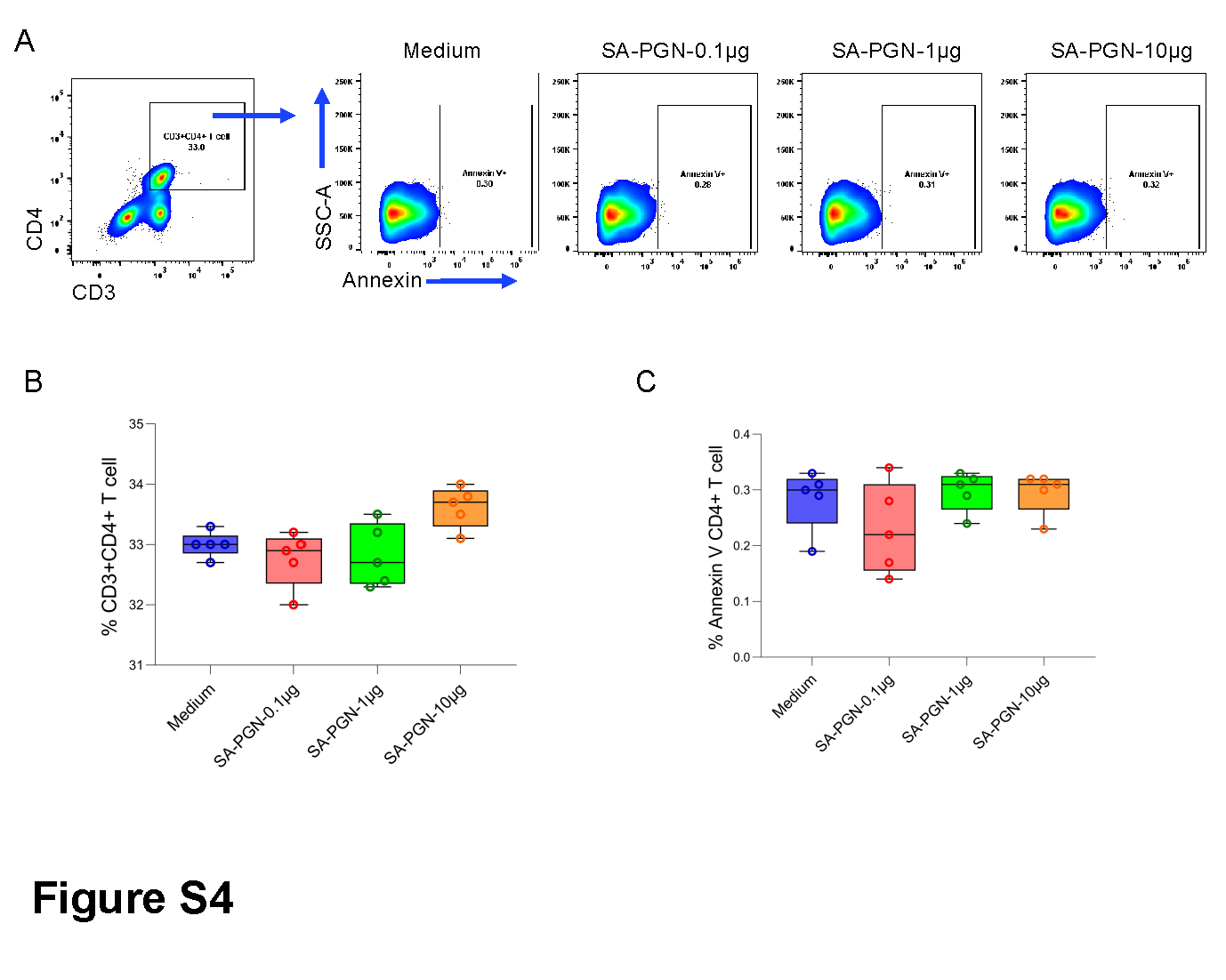

Supplement: Figure S4 — S. aureus PGN does not directly induce gut CD4+ T cell apoptosis in vitro. [file jvi.01965-25-s0005.tiff]

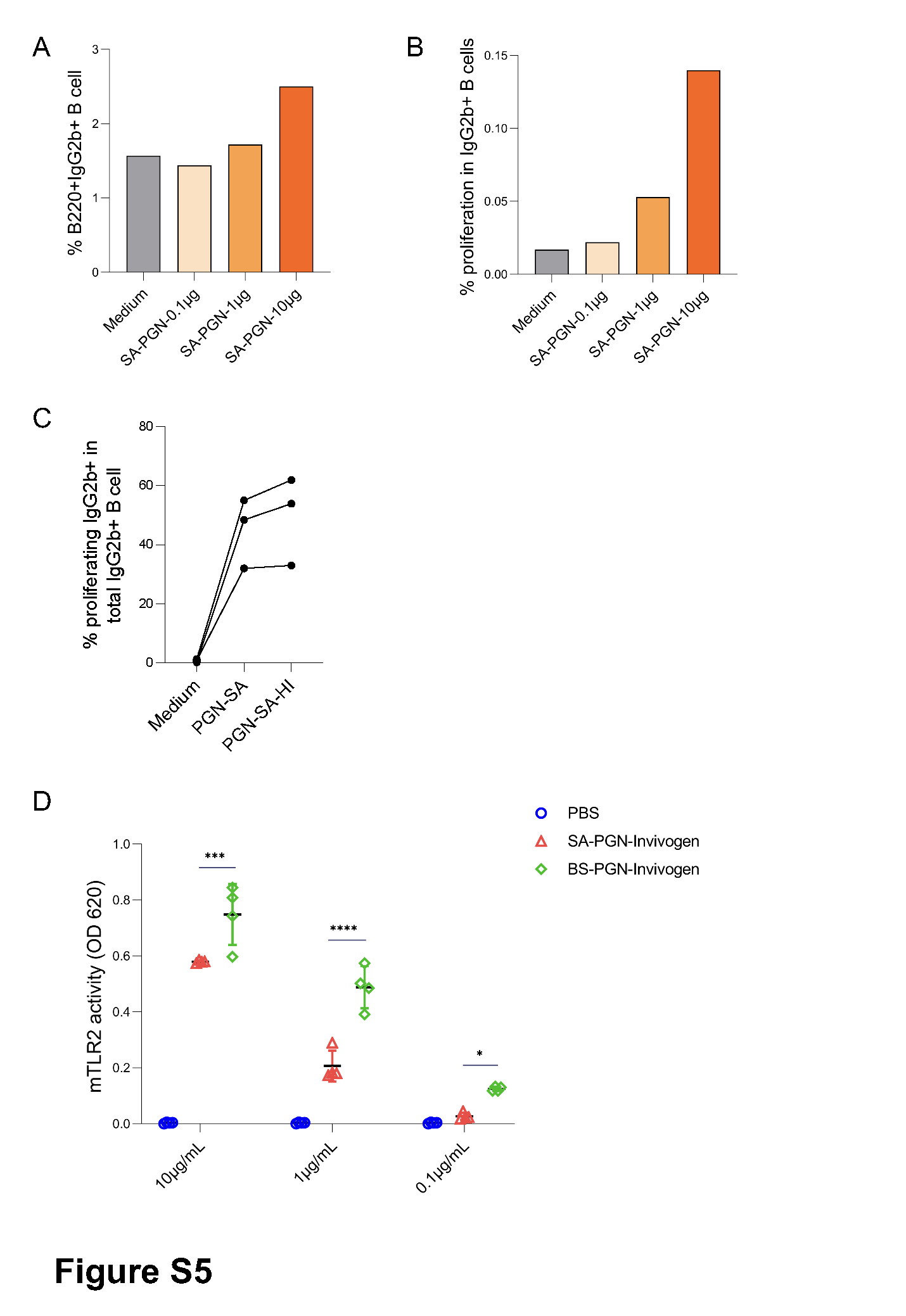

Supplement: Figure S5 — PGN dose titration for CSR and TLR2 activity. [file jvi.01965-25-s0006.tiff]

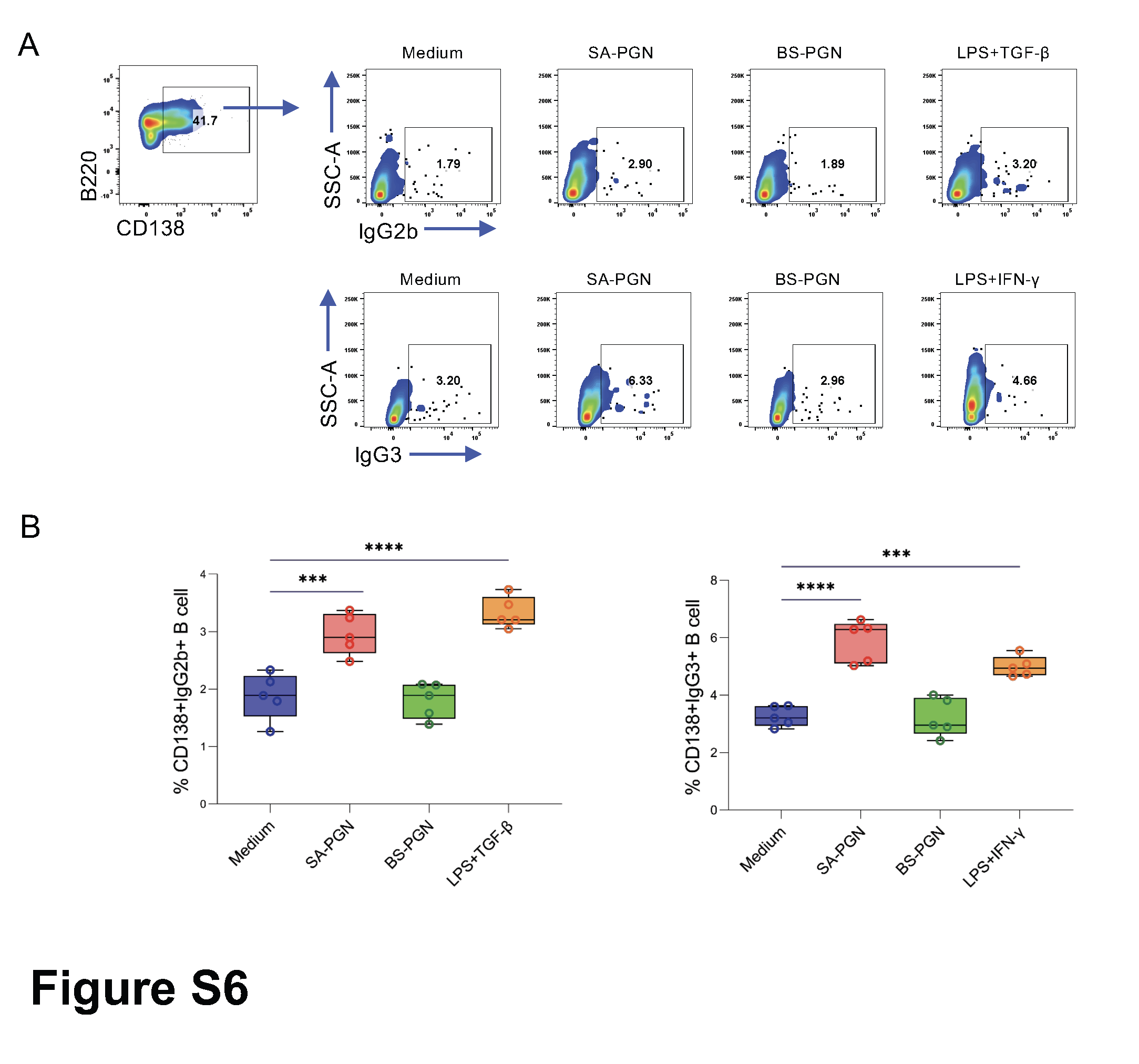

Supplement: Figure S6 — S. aureus PGN induces CSR in vitro. [file jvi.01965-25-s0007.tiff]
