# Supplementary material for: Systemic translocation of Staphylococcus aureus promotes autoimmunity: implications in autoantibody-mediated poor immune reconstitution from antiretroviral therapy in HIV
Source: J Virol. 2026 Apr 3;100(5):e01965-25. doi: 10.1128/jvi.01965-25 (PMC13185582; doi:10.1128/jvi.01965-25)
Supplement: Supplemental legends — Descriptive legends for Fig. S1 to S6. [file jvi.01965-25-s0008.docx]

**Figure S1**. **Elevated plasma anti-CD4 IgG1 and IgG3 subclasses in PWH on ART**. (A) The median plasma levels of anti-CD4 IgG, IgM, and IgA in controls (n = 15) and HIV+/ART+ subjects (n = 23). (B) The median plasma levels of anti-CD4 IgG subclasses (IgG1-IgG4) between the two groups. Mann-Whitney tests.

**Figure S2. Confirmation of established EcoHIV infection in mice.** The EcoHIV virus was verified by qPCR in blood samples collected one month after infection.

**Figure S3. S. aureus PGN does not affect gut CD4−CD3+ T cells in vivo.** (A) Gating strategy. (B) Percentages of CD4−CD3+ T cells among total CD3+ T cells and IgG+CD4−CD3+ T cells in mesenteric lymph nodes. ANOVA.

**Figure S4. S. aureus PGN does not directly induce gut CD4+ T cell apoptosis in vitro.** Lymphocytes from mesenteric lymph nodes were treated with S. aureus PGN (0.1–10 μg/mL) for 4 days. (A-B) Percentages of CD4+ T cells and Annexin V+CD4+ T cells. ANOVA.

**Figure S5. PGN dose titration for CSR and TLR2 activity.** (A–B) S. aureus PGN induced total (A) and proliferating (B) IgG2b+ B cells. (C) CSR induction by heat-inactivated vs. native S. aureus PGN. (D) mTLR2 reporter cell responses after 16 h treatment. ANOVA.

**Figure S6. S. aureus PGN induces CSR in vitro.** Splenic B cells from naive mice were cultured with PGN (10 μg/mL) for 96 h. Percentages of CD138+ IgG2b+ and IgG3+ plasmablasts (%CD138+CFSE^lowIgG2b/IgG3+ in B220+ cells) are shown (A–B). ANOVA.
